# Editorial: The role of angiogenesis and immune response in tumor microenvironment of solid tumor

**DOI:** 10.3389/fimmu.2023.1195390

**Published:** 2023-04-18

**Authors:** Zichao Guo, Yimei Jiang, Baochi Ou, Xin Lu, Xi Cheng, Ren Zhao

**Affiliations:** ^1^ Department of General Surgery, Ruijin Hospital, Shanghai Jiao Tong University School of Medicine, Shanghai, China; ^2^ Shanghai Institute of Digestive Surgery, Ruijin Hospital, Shanghai Jiao Tong University School of Medicine, Shanghai, China; ^3^ Department of General Surgery, First Affiliated Hospital of Anhui Medical University, Hefei, China; ^4^ Department of Biological Sciences, University of Notre Dame, Notre Dame, IN, United States

**Keywords:** tumor microenvironment, tumor immunotherapy, angiogenesis and antiangiogenetic therapy, tumor immune infiltration, tumor treatment and prognostic prediction

The microenvironment of solid tumors consists of unrestricted proliferating tumor cells, cancer-associated fibroblasts (CAFs), stroma cells, immune infiltrations, and blood vessels. It is generally recognized that immune system has involved in initializing tumorigenesis, regulating tumor progression, and affecting the effect of tumor therapy (1–4). Within the tumor microenvironment, immune infiltrations and the formation of neoplastic neovascularization are critical for supporting tumor progression (5–8). In recent years, specific genes and molecular patterns, associated with immune response and angiogenesis, have been discovered as predictors of tumor treatment and patients’ prognosis, and as targets of tumor immunotherapy and combined therapy (9–12). Under the circumstance that both antiangiogenetic therapy and immunotherapy have renovated tumor treatment and inspired medical explanation, this Research Topic collected 15 scientific studies focused on tumor immune microenvironment, tumor immunotherapy and antiangiogenetic therapy.

Angiogenesis is considered a significant event in the tumor microenvironment that affects immune infiltrations. Li et al. disclosed the important role of N6-methyladenosine (m6A) modification in synergizing with complex perivascular pathological ecology to mediate the immunosuppressive tumor microenvironment in low-grade glioma (LGG). By means of bioinformatic analysis, the authors elucidated the relevance of the angiogenesis-related genes and m6A regulators (MAGs), and established a high-performance gene-signature (MASig) that revealed somatic mutational mechanisms by which MAGs affect the sensitivity to treatment in LGG patients. This study provided a novel strategy for supporting the development of precision diagnosis and treatment on patients with LGG. As a novel selective vascular endothelial growth factor receptor 2 inhibitor, apatinib expanded the scope of antiangiogenetic therapy. Li et al. overviewed the pharmacological properties of apatinib, relevant molecular mechanisms, and its latest clinical applications. The authors shed lights on the monotherapy and combined application of apatinib with immunotherapy on patients with advanced, chemotherapy-refractory digestive system cancer.

Within the tumor microenvironment, residing microbiota emerge as a research hot spot in immune response. Xu et al. discovered that *Actinomyces* and *Schaalia cardiffensis* were the essential microbiota in the sporadic young-onset colorectal cancer (CRC) group. The *Actinomyces* in CRC was found to be co-localized with CAFs and activated the TLR2/NF-κB pathway and reduces CD8+ T lymphocyte infiltration in CRC microenvironment, providing a potential promising non-invasive tool and intervention target for anti-tumor therapy. Dazzling interactions within the tumor microenvironment raise various explorations in gene patterns of solid tumors. Shen et al. revealed that the expression levels of ETV1 and ETV5 are positively associated with the poor prognosis of CRC patients. Based on multiple bioinformatic databases and a single cohort, the intersections of various analysis highlighted ETV1 as a significant convergence of tumor progression and immune infiltrations, especially CAFs and M2 tumor-associated macrophages (TAMs), hence providing a potential target for CRC treatment. Cao et al. took an in-depth look at the effect of mitochondrial energy metabolism pathway-related genes (MMRGs) on tumor progression, tumor immunity and prognosis of patients with colon adenocarcinoma (COAD). Innovatively, the authors proposed a novel risk model and different COAD subtypes characterized by MMRG patterns, which prompts classification of patients with different prognosis. In addition, Yu et al. identified SPP1-positive macrophages might correlate with cell senescence and lead to poor prognosis of CRC. They presented a novel prognosis model based on senescence-related genes which reflected immunosuppressive tumor microenvironment. Wang et al. identified 34 insertion genes as oxidative stress- and ferroptosis-related genes (OFRG) and developed an OFRG-related prognostic signature to predict the prognosis and therapeutic response in patients with CRC. The study provided a novel tool to precisely distinguish cold and hot tumors in CRC on gene expression pattern level. Li et al. revealed a panoramic view of tumor-related inflammatory cytokines of CRC. Differential expression patterns of cytokines in peripheral blood were related to distant metastasis and the size of the primary tumor, prompting chemokines and cytokines may be potential prognostic factors and targets for treatment. Weng et al. focused on the epithelial-to-mesenchymal transition related gene alternative splicing (AS) event in pancreatic ductal adenocarcinoma (PDAC). The study found that AS events of TMC7 and CHECK1 were associated with liver metastasis in PDAC, especially exon 17 of TMC7 could be a potential therapeutic target in PDAC. Sang et al. came up with a novel gastric cancer (GC) prognostic model and a GC immune stratification based on the tumor microenvironment gene expression patterns from an individual GC cohort. Connective tissue growth factor was reported to be overexpressed in GC and exhibited a significant correlation with the abundance of fibroblasts, potentially related to CAFs. This study provided a novel perspective from tumor microenvironment to further explore carcinogenesis and tumor treatment. Xu et al. emphasized FOXP2 as an immune regulatory factor in thyroid cancer. From the macro perspective, the authors revealed that FOXP2 may affect tumor microenvironment and immune infiltrations, thus providing a new potential diagnostic and prognostic marker of thyroid cancer.

To shape the tumor microenvironment, and revert the immune desert phenotype of malignancy for anti-tumoral immune augmentation, researchers exhibit various explorations. Han et al. comprehensively reviewed the application of ultrasound-target microbubble destruction treatment in adjuvant tumor immunotherapy and its effect on tumor microenvironment modulation, especially on tumor angiogenesis and shaping immune infiltrations. Zhao et al. proposed a novel combination of losartan and doxorubicin hydrochloride liposome (Dox-L) in assisting anti-programmed cell death-1 immunotherapy treating triple-negative breast cancer (TNBC). The authors revealed that losartan could deplete extracellular matrix and facilitate the delivery of Dox-L, and by which the combined therapy was characterized as promoting dendritic cell maturation and reprogramming M2 TAMs to M1 TAMs, thus normalizing immunological-cold tumor microenvironment of TNBC to optimize the immunotherapy. Rahmy et al. developed a beta-90kD heat shock protein selective inhibitor NDNB1182 that induces cytotoxicity to cancer cells rather than causing heat shock response. By means of mouse models of prostate cancer and breast cancer, the authors proved that NDNB1182 collaborated with immune checkpoint blockade therapy, exhibited a synergistic anti-tumoral effect, and showed superior tolerability to pan-Hsp90 inhibition. In this journey, cancer vaccine is unique in tumor prevention and treatment. Jia et al. summarized different categories of CRC vaccines and demonstrated the current scenarios of relevant clinical trials. Especially, they focused on the latest advancements of CRC nano-vaccines and neoantigen vaccines, providing the trends in CRC vaccine development.

In summary, the studies included in this Research Topic exhibit the latest advances in angiogenesis and immune response in the tumor environment of solid tumor. We would like to express our sincere gratitude to all authors, reviewers, editors, topic editors and the editorial team of *Frontiers in Immunology* for their devotion and assistance in the process of reviewing and publishing all these studies in this Research Topic. In-depth explorations in tumor immune microenvironment and tumor antiangiogenetic therapy promises to move researches in tumorigenesis, tumor progression and tumor treatment a step forward in the next decade.

## Author contributions

All authors listed have made a substantial, direct, and intellectual contribution to the work and approved it for publication.

## References

[1] Balkwill F Mantovani A . Inflammation and cancer: back to virchow? Lancet (2001) 357:539–45. doi: 10.1016/S0140-6736(00)04046-0 11229684

[2] Fridman WH Pagès F Sautès-Fridman C Galon J . The immune contexture in human tumours: impact on clinical outcome. Nat Rev Cancer (2012) 12:298–306. doi: 10.1038/nrc3245 22419253

[3] Feng H Guo Z Chen X Liu K Li H Jia W . Excessive HSP70/TLR2 activation leads to remodeling of the tumor immune microenvironment to resist chemotherapy sensitivity of mFOLFOX in colorectal cancer. Clin Immunol (2022) 245:109157. doi: 10.1016/j.clim.2022.109157 36244673

[4] Shankaran V Ikeda H Bruce AT White JM Swanson PE Old LJ . IFNgamma and lymphocytes prevent primary tumour development and shape tumour immunogenicity. Nature (2001) 410:1107–11. doi: 10.1038/35074122 11323675

[5] Liu Y Cao X . Characteristics and significance of the pre-metastatic niche. Cancer Cell (2016) 30:668–81. doi: 10.1016/j.ccell.2016.09.011 27846389

[6] Cassetta L Pollard JW . Targeting macrophages: therapeutic approaches in cancer. Nat Rev Drug Discov (2018) 17:887–904. doi: 10.1038/nrd.2018.169 30361552

[7] Fukumura D Kloepper J Amoozgar Z Duda DG Jain RK . Enhancing cancer immunotherapy using antiangiogenics: opportunities and challenges. Nat Rev Clin Oncol (2018) 15:325–40. doi: 10.1038/nrclinonc.2018.29 PMC5921900 29508855

[8] Qian B-Z Pollard JW . Macrophage diversity enhances tumor progression and metastasis. Cell (2010) 141:39–51. doi: 10.1016/j.cell.2010.03.014 20371344 PMC4994190

[9] Marin-Acevedo JA Kimbrough EO Lou Y . Next generation of immune checkpoint inhibitors and beyond. J Hematol Oncol (2021) 14:45. doi: 10.1186/s13045-021-01056-8 33741032 PMC7977302

[10] Galluzzi L Humeau J Buqué A Zitvogel L Kroemer G . Immunostimulation with chemotherapy in the era of immune checkpoint inhibitors. Nat Rev Clin Oncol (2020) 17:725–41. doi: 10.1038/s41571-020-0413-z 32760014

[11] Lan C Shen J Wang Y Li J Liu Z He M . Camrelizumab plus apatinib in patients with advanced cervical cancer (CLAP): a multicenter, open-label, single-arm, phase II trial. J Clin Oncol (2020) 38:4095–106. doi: 10.1200/JCO.20.01920 PMC7768345 33052760

[12] Rahma OE Hodi FS . The intersection between tumor angiogenesis and immune suppression. Clin Cancer Res (2019) 25:5449–57. doi: 10.1158/1078-0432.CCR-18-1543 30944124

